# Temporal Discounting of Money and Face Masks During the COVID-19 Pandemic: The Role of Hoarding Level

**DOI:** 10.3389/fpsyg.2021.642102

**Published:** 2021-06-09

**Authors:** Loreta Cannito, Stefano Anzani, Alessandro Bortolotti, Rocco Palumbo, Irene Ceccato, Adolfo Di Crosta, Alberto Di Domenico, Riccardo Palumbo

**Affiliations:** ^1^Department of Psychological Sciences, Health and Territory, University “G. D'Annunzio” of Chieti-Pescara, Chieti Scalo, Italy; ^2^Center for Advanced Studies and Technology, University “G. D'Annunzio” of Chieti-Pescara, Chieti Scalo, Italy; ^3^Department of Neuroscience, Imaging and Clinical Sciences, University “G. D'Annunzio” of Chieti-Pescara, Chieti Scalo, Italy

**Keywords:** temporal discount, impulsivity, hoarding, surgical masks, COVID-19, discount rate, consumer behavior

## Abstract

The current study examines the association of individual hoarding levels with temporal discounting of different commodities during the COVID-19 pandemic. Based on their hoarding level, participants were assigned to the Hoarding Group (HG) or the Non-Hoarding Group (NHG). Participants performed two delay discounting tasks: a traditional task with monetary options and a modified task, where money was replaced with disposable surgical masks, a needed commodity during the pandemic. Results revealed a stronger preference for immediate commodity, therefore a higher discount rate, when evaluating surgical masks compared to money in the whole sample, and an overall higher tendency in discounting both type of rewards in the NHG compared to the HG. Moreover, non-hoarders discounted money significantly more than hoarders, while no significant differences were detected in the surgical mask version of the task. Possible explanations for this result are discussed in the light of a situational frame that makes salient the notion of scarcity, like the one induced by the COVID-19 pandemic. The hoarding dimension of cluttering was found to be the only dimension to significantly correlate with the discount rate on surgical masks. Altogether, these findings shed light on the role of general hoarding level and specific hoarding dimensions on intertemporal preferences with different commodities by contributing to the theoretical debate about impulsivity in hoarders' behavior. Furthermore, the present results help to understand the general population's preferences during times of crisis, thus contributing to the investigation of the effects of COVID-19 on consumers' behavior.

## Introduction

At the beginning of 2020, a new virus began to spread worldwide, threatening people's physical health, so that on March 11^th^, the World Health Organization (WHO) declared the COVID-19 a pandemic. This situation has provoked several consequences on people's mental health because of reasons directly connected to the virus spread, such as fear or anxiety to be infected or grief over the death of a relative or friend (e.g., Cannito et al., 2020; Di Crosta et al., 2020), but also depression associated to loneliness and social distancing due to governments' decisions of implementing social isolation (e.g., Palgi et al., 2020). Among other life's domains, buying habits are likely to have been influenced by the situation since it has already been shown that, in similar times of crisis (e.g., earthquakes, hurricanes, wars, and so on), people tend to manifest herd behavior and hoarding when facing a mass threat. Hoarding behavior is defined as the act of collecting and safeguarding a larger number of possessions than the one needed for the future (Chu, 2018). From an economic and social perspective, the impact of over-acquiring products during a crisis represents a problem for the supply chain disruption risk management. The assumption that people show more hoarding behavior in times of crisis has been widely documented in the past. For example, in association with the Tohoku earthquake in 2011, Hori and Iwamoto, by identifying specific hoarders' profiles, reported how people changed their buying habits in the direction of increased hoarding (Hori and Iwamoto, 2014). Similarly, it has been evidenced that seasonal goods are systematically hoarded during every typhoon season in Taiwan (Zanna and Rempel, 1988). Furthermore, attitude to risk-driven hoarding and disaster-induced affective response are key factors that impact the agricultural-food supply chain during this season (Sheu and Kuo, 2020). Given this past evidence, it is not surprising that, since the beginning of the COVID-19 pandemic emergency, we witnessed hoarding of specific supplies all over the world (e.g., toilet paper; sanitizing gel, etc.) both directly connected or not to the COVID-19 (e.g., Kirk and Rifkin, 2020). Italy was one of the very first countries to experience the direct and indirect consequences of the pandemic. From the consumer behavior's perspective, during the initial phase of the first pandemic wave (approximately between March and April 2020) and while the whole nation was dealing with a total lock-down, stores have been systematically emptied of personal protective equipment for coronavirus disease such as surgical masks or sanitizing gel, due to fear of shortage or disruption in availability, and even of products that were not in short supply. The phenomenon is not new for the psychology of unavailability. Since its first formulation, in Brock's commodity theory (Brock, 1968), the notion that scarcity “enhances the value of everything that can be possessed” has been widely confirmed (e.g., Verhallen and Robben, 1994) and applied for marketing purposes (e.g., Brannon and Brock, 2001). In the commodity theory, the definition of scarcity includes (1) limits on the supply or the number of suppliers; (2) restrictions limiting possession of a commodity; (3) delays in providing a commodity; and (4) cost of acquiring or keeping or of providing a commodity (Brock, 1968). Therefore, as a possible explanation for consumers' reaction at the beginning of the pandemic, people may have anticipated imminent product scarcity and decided to hoard and stock supplies as a future-oriented planning strategy. This behavior would be in line with evidence suggesting that, when a threat is perceived, hoarding is activated as an evolutionary-based mechanism driven by the fear of being caught not prepared for the situation. For example, it has been documented that the hoarding level is related to the uncertainty of future product availability (Byun and Sternquist, 2012). At the same time, hoarding is enhanced after a negative event because of augmented anticipated regret and risk aversion (Frost and Gross, 1993; Cameron and Shah, 2015; Gupta and Gentry, 2019). Following this view (real or perceived), scarcity activates a future-oriented mindset due to the fear of being unprepared, and hoarding represents the coping mechanism to (a) reduce the unpleasure associated with the emotion of fear, and (b) solve the ultimate problem (not to be caught unprepared). On the other hand, it is possible that due to the lock-down, people may have experienced a constriction in the opportunity to buy products and therefore reacted to this perceived loss of freedom and control (Lynn, 1991) by hoarding and stocking supplies, as suggested by psychological reactance theory (Clee and Wicklund, 1980). Hence, to hoard threatened goods can reduce this feeling of loss of control (Frost and Hartl, 1996). Overall, based on these theoretical approaches, hoarding may either be linked to future planning and to the need to be prepared, or it could be explained by consumers' engagement in reactive emotional response as suggested by psychological reactance theory.

However, it should be noted that, for some individuals, there are internal factors that may further promote hoarding behavior during times of crisis. It is the case of people suffering from Hoarding Disorder (HD), defined as “persistent difficulty discarding or parting with possessions, regardless of their actual value.” Just recently, HD has become a diagnostic disorder of its own, belonging to the disorder class of Obsessive-Compulsive and Related Disorders from the DSM-V (DSM-5, American Psychiatric Association, 2013). The difficulty in discarding/parting results in the accumulation of objects that, in turn, prevents the use of living areas for their intended use (a phenomenon known as cluttering) and causes significant distress or impairment in social, occupational, or other important areas of functioning (DSM-5, American Psychiatric Association, 2013). Five hoarding dimensions have been identified: cluttering, excessive acquisition, difficult discarding, distress, and impairment (e.g., Tolin et al., 2010). Hoarding Disorder, at the moment, can be diagnosed with or without the specifier of “excessive acquisition” even if there is a large number of studies that highlight how the majority of people with HD also exceed in buying/acquisition and that the presence of this behavior is associated with a more severe clinical picture (Frost and Muller, 2014; Tompkins, 2015; Timpano et al., 2016; Thompson et al., 2017). The notion that excessive acquisition (together with difficulty discarding) is a key factor in HD is additionally supported by evidence about the relationship between HD and Compulsive Buying (CB), traditionally framed as an impulse control disorder. For example, it has been reported that among hoarders, 61% met the criteria for a diagnosis of CB (Frost et al., 2002). More severe CB was also detected in compulsive buyers with comorbid hoarding (Mueller et al., 2007). Altogether these findings suggest that excessive acquisition, together with difficulty discarding, is a central dimension of hoarding behavior. By the way, the cause of excessive acquisition in hoarding is still unclear. One proposed account relies on the existing link between hoarding and impulsivity domain (e.g., Timpano et al., 2013). Timpano et al. (2013) reported that specific facets of impulsivity, namely motor impulsivity, attentional impulsivity, lack of perseverance, and urgency (i.e., impulsive response to negative affect), were strongly associated with hoarding symptoms. On the contrary, another research highlighted that the link between some hoarding dimensions and impulsivity aspects disappeared when controlling for participants' age (Rasmussen et al., 2013). Recently, Laato et al. (2020) found a strong link between intention to self-isolate during pandemic and intention to hoard and make unusual purchases. Therefore, the authors suggested that consumer behavior is directly connected to the anticipated time spent in self-isolation (Laato et al., 2020). This finding contributes to disconfirm the impulsivity-based pathogenesis for HD while suggesting a future planning prompting aim. Hence, hoarders do not always show the typical features of those psychopathological conditions that have been traditionally investigated and treated by focusing on impulsivity/impulse control, such as short-sighted decision making (Vickers et al., 2016). For example, Vickers et al. (2016) reported that hoarders, compared to non-hoarders, were more impulsive (more impatient) for consumables rather than for money, as measured through a temporal (or delay) discounting task. Temporal discounting is a cognitive phenomenon defined as the progressive decline in the subjective value of a given reward as the time of its receipt is delayed in time. In other words, temporal discounting refers to the tendency to prefer a smaller reward immediately available, instead of a larger reward, but for which a waiting time is required. In literature, the two options are referred to as “impulsive choice” and “self-controlled choice,” respectively (e.g., Odum, 2011). This phenomenon has received increasing attention in the last years as it has been shown that temporal discounting predicts suboptimal behavior in several domains, such as health and food style, finances, household savings, and personal development (e.g., Snider et al., 2019). Furthermore, prior studies have demonstrated how steep delay discounting is associated with several dysfunctional behaviors or clinical conditions such as smoking (e.g., Audrain-McGovern et al., 2009), drug abuse (e.g., Kirby et al., 1999), gambling (e.g., Calluso et al., 2020), and CB (e.g., Williams, 2012). It is against this background that excessive discounting has been a candidate as a trans-disease process for those conditions that share an inability to delay a gratification leading to a “here and now” bias (e.g., Bickel et al., 2012; Amlung et al., 2019). Crucially, hoarders showed no “here and now” bias for money (Vickers et al., 2016). This result appears to be consistent with the phenomenology of HD that does not seem to be driven by the inability to wait, but rather by a specific desire to collect goods as a proxy to control future needs.

Based on these findings, we aimed to answer the following questions:

1. Do hoarders and non-hoarders differently discount money and scarcely available commodities such as disposable surgical masks (a personal protective equipment from coronavirus disease)?
2. Are the discount rates applied to monetary reward (an indicator of implicitly measured impulsivity) and surgical masks reward (an indicator of consumer's preference toward scarcely available commodity) correlated?

The surgical mask was chosen as a commodity to compare with money as it was known at the time to be a protective equipment for preventing the spread of COVID-19 and scarcely available. Unlike other goods in shortage (for example, some food products), surgical masks are not perishable (a quality that could have influenced the selection of future alternatives).

## Materials and Methods

Participants were recruited through a public announcement online and provided informed consent following the Declaration of Helsinki's ethical standards. The research protocol was approved by the Institutional Review Board of Psychology (IRBP) of the Department of Psychological, Health and Territorial Sciences at G. D'Annunzio University of Chieti-Pescara. All participants had no previous history of psychiatric disorders. The whole procedure was administered during the last week of April 2020 in web-based form through Qualtrics software (qualtrics.com) due to the impossibility to invite participants in the laboratory given the ongoing lock-down in Italy. Participants received no monetary or other forms of compensation for their participation.

### Participants

We kept recruiting potential participants until we obtained a sample of 50 individuals whose HRS-I total score was >14 (cut-off score; Tolin et al., 2010), hereafter Hoarding Group (HG). We got this attainment after 198 screened participants. A Non-Hoarding Group (NHG) was then created by selecting the 50 participants with the lowest HRS-I total scores from the remaining participants, matched for gender, age and educational level to participants in the HG. Selected participants for both groups (*N* = 100) were contacted a second time to complete the Temporal Discounting Task. Descriptive statistics for both groups are reported in Table 1.

**Table 1:** Descriptive statistics for age, Hoarding Rating Scale-Interview (HRS-I) scores, and time estimation scores.

	**Group**	
	**Hoarding**	**Non-hoarding**
**Age**	24.8 (5.11)	31.2 (13.5)
**Gender**		
Male	*N* = 5	*N* = 3
Female	*N* = 45	*N* = 47
**Education**		
Secondary school	*N* = 2	*N* = 1
Upper secondary school	*N* = 13	*N* = 16
University college or higher	*N* = 35	*N* = 33
**Cluttering**	1.24 (0.47)	3.28 (1.59)
**Difficult discarding**	1.44 (0.70)	4.58 (2.07)
**Excessive acquisition**	1.34 (0.65)	3.56 (1.58)
**Distress**	1.16 (0.42)	3.94 (1.86)
**Impairment**	1.08 (0.34)	3.24 (1.73)
**Total HRS-I**	6.26 (1.25)	18.6 (3.73)
**Time to return to normal (in months)**	10.4 (6.3)	12.3 (7.7)

*Mean (SD) separate for hoarding groups*.

### Hoarding Rating Scale-Interview (HRS-I)

As the first step, we conducted a preliminary assessment by asking participants to complete the Italian version of the Hoarding Rating Scale-Interview, which has been shown to have high internal consistency, strong correlation with other measures of hoarding, and the ability to discern hoarders and non-hoarders (HRS-I; Tolin et al., 2010; Faraci et al., 2019). The scale is a self-report measure based on five items addressing five different dimensions: cluttering, difficult discarding, acquisition, distress, and impairment. Each item is rated on a 9-point Likert scale from 0 (“none”) to 8 (“extreme”). As in a previous study, no differences were found between hoarders and non-hoarders regarding gender, age, marital status, level of education, and employment status (Bulli et al., 2014). None of these variables were considered as inclusion/exclusion criteria.

### Temporal Discounting Task

Participants' intertemporal preferences toward money and surgical face masks were assessed using the extensively validated and commonly used 27-item MCQ—Monetary Choice Questionnaire (Kirby et al., 1999). This delay discounting task was presented both in a traditional (hereafter money) version and in a modified (hereafter surgical mask) version. In the money version, on each of the 27 choice trials, participants were shown a couple of monetary rewards and asked to choose between the immediately available but smaller alternative (e.g., €25 today) and the delayed but larger one (e.g., €35 in 25 days). In the surgical mask version, the immediate and delayed monetary reward amounts were converted in numbers of available surgical masks by applying €1 apiece value. This conversion rule was established based on the approximated mean price for surgical masks in Italy during the data collection period. Thus, in the surgical mask version, participants were asked to express a preference between a smaller number of surgical masks immediately available (e.g., 25 masks today) and more surgical masks available only after a waiting time (e.g., 35 surgical masks in 25 days). Thus, each participant completed a total of 54 intertemporal choices (27 in the money version of the task and 27 in the mask version of the task). The participant sample was divided in half, with half completing the money version as the first task and the surgical mask version as the second task. The other half completed the tasks in the reverse order (see Supplementary Material File, Tables S1, S2).

### Time Estimation

After tasks completion, participants were asked to indicate in how many months, in their opinion, we would be able to return to “normal life,” where “normal” referred to being able to do everything they did before the pandemic began. This variable was assessed as it could have influenced intertemporal preferences in the surgical mask version of the task, as suggested by Laato et al. (2020) findings.

### Behavioral Analysis

Based on the participants' observed preferences in the money and surgical masks versions of the temporal discounting task, we calculated the discount rate, i.e., *k* score, using an R syntax (Gray et al., 2016). Discounting rates were calculated using Mazur's (1987) and Kirby et al.'s (1999) hyperbolic discounting Equation (1). The equation contains a single free parameter, which is interpreted as the degree of delay (*k*) discounting, i.e., discount rate. When the *k* value increases, the subjective value of the delayed alternative is more steeply discounted:

(1) $$\begin{array}{r} V=A/\left(1+kD\right) \end{array}$$

*V* is the present value of the delayed reward, *A* is the amount of the delayed reward, *D* is the delay, and *k* is the individual discount rate. Therefore, *k* describes the steepness of the discounting curve or, in other words, the degree to which a value is devalued over time. Following this procedure, we obtained an individual *k* value. A larger value of *k* indicates a steeper discounting of the delayed reward.

Furthermore, each one of the 27 trials has been classified according to its *k* rank. The *k* rank classifies items into nine different groups and is defined based on *k* indifference. *k* indifference is the value of the discount rate at which the immediate and delayed rewards are of equal value according to Equation (1). Each of the nine different groups includes three items defined as small, medium, and large based on the Later Delayed Reward (LDR) size. Thus, we obtained four different *k* scores (small, medium, large, and an overall score). The obtained *k* scores were then log-transformed to ensure normality.

## Results

### Effects of Hoarding Level on Discount Rates

**Figure 1:**
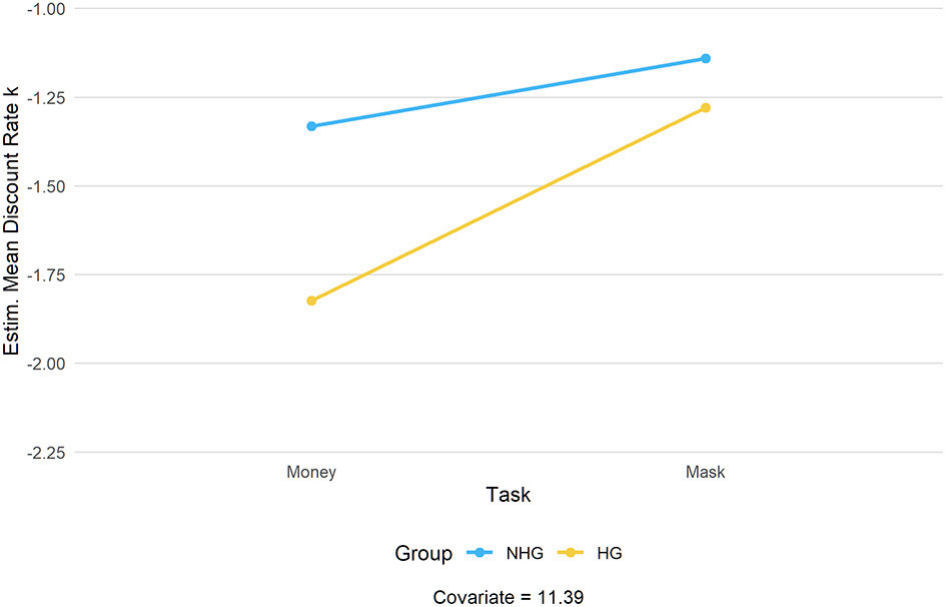
Estimated mean discount rate across task versions (money vs. surgical mask) and hoarding group (HG vs. NHG).

A mixed model analysis of covariance (ANCOVAs) 2 (Task Version: money, surgical mask) × 2 (Group: HG, NHG) was performed to examine differences in the discount rate (*k*) within the tasks and between HG and NHG, after controlling for time estimation scores (covariate). Results are reported in Figure 1 and Table 2. The assumptions of normality, homoscedasticity, outliers, and homogeneity of regression slopes were assessed. The sphericity assumption does not apply when there are only two repeated measurements. Normality was evaluated using a Q–Q scatterplot, which compares the residuals' distribution with the normal distribution. Homoscedasticity was assessed through Levene's test which confirmed homogeneity of variances for *k*_money_ [*F*_(1, 98)_ = 0.213, *p* > 0.05] and for *k*_masks_ [*F*_(1, 98)_ = 1.07, *p* > 0.05]. To identify influential points in the residuals, Mahalanobis distances were computed and compared to a χ^2^ distribution. No outliers were detected. The assumption of homogeneity of regression slopes was assessed by running the mixed model ANCOVA but including the interaction term between the independent variable and the covariate. The interaction was not significant; therefore, the assumption was met.

**Table 2:** Results of the mixed ANCOVA analyses with Task version (money, surgical masks) and Group (HG, NHG) effects on discount rate (*k*) based on small, medium, large, or overall later delayed reward (LDR) size.

**Effect**	**LDR size**	***F*_**(1, 97)**_**	***p***	**η*_***p***_*^**2**^**
Task version	Overall	13.97	**0.000**	0.126
	Small	5.86	**0.017**	0.057
	Medium	12.03	**0.001**	0.11
	Large	15.22	**0.000**	0.136
Group	Overall	20.83	**0.000**	0.177
	Small	12.15	**0.001**	0.111
	Medium	20.60	**0.000**	0.175
	Large	21.34	**0.000**	0.180
Task version × group	Overall	8.51	**0.004**	0.081
	Small	6.96	**0.010**	0.067
	Medium	4.21	**0.043**	0.042
	Large	5.98	**0.016**	0.058
Covariate	Overall	4.24	**0.042**	0.042
	Small	2.29	0.133	0.023
	Medium	3.14	0.079	0.031
	Large	4.38	**0.039**	0.043
Task version × covariate	Overall	0.375	0.542	0.004
	Small	0.001	0.982	0.000
	Medium	0.337	0.563	0.003
	Large	0.380	0.539	0.004

*Significant p-values are reported in bold*.

ANCOVA results showed that the overall discount rate (*k*) was significantly predicted by time estimation scores, *F*_(1, 97)_ = 4.24, *p* = 0.042, η_*p*_^2^ = 0.04. After accounting for the effect of the covariate, the group factor was significant, *F*_(1, 97)_ = 20.83, *p* < 0.001, η_*p*_^2^ = 0.18, with NHG participants (*M* = −1.236, *SD* = 0.049) discounting the delayed alternative significantly more than HG participants (*M* = −1.552, *SD* = 0.049). This shows there is an overall observed higher farsightedness in the hoarding compared to the NHG. A main effect of task version emerged, *F*_(1, 97)_ = 13.99, *p* < 0.001, η_*p*_^2^ = 0.13, with all participants discounting money (*M* = −1.578, *SD* = 0.039) significantly less than surgical masks (*M* = −1.210, *SD* = 0.051). Finally, we found a significant interaction effect between task version and group, *F*_(1, 97)_ = 8.51, *p* < 0.01 η_*p*_^2^ = 0.08, indicating that the discount rate's difference between the two tasks was larger for the HG (*M*_money_ = −1.82, *SE*_money_ = 0.05; *M*_mask_ = −1.28, *SE*_mask_ = 0.07, *p* < 0.001) rather than for the NHG (*M*_money_ = −1.33, *SE*_money_ = 0.05; *M*_mask_ = −1.14, *SE*_mask_ = 0.07, *p* < 0.05) (see Figure 1). No significant interaction effect between task version and time estimation scores was detected, *F*_(1, 97)_ = 0.375, *p* > 0.05, η_*p*_^2^ = 0.004.

**Figure 2:**
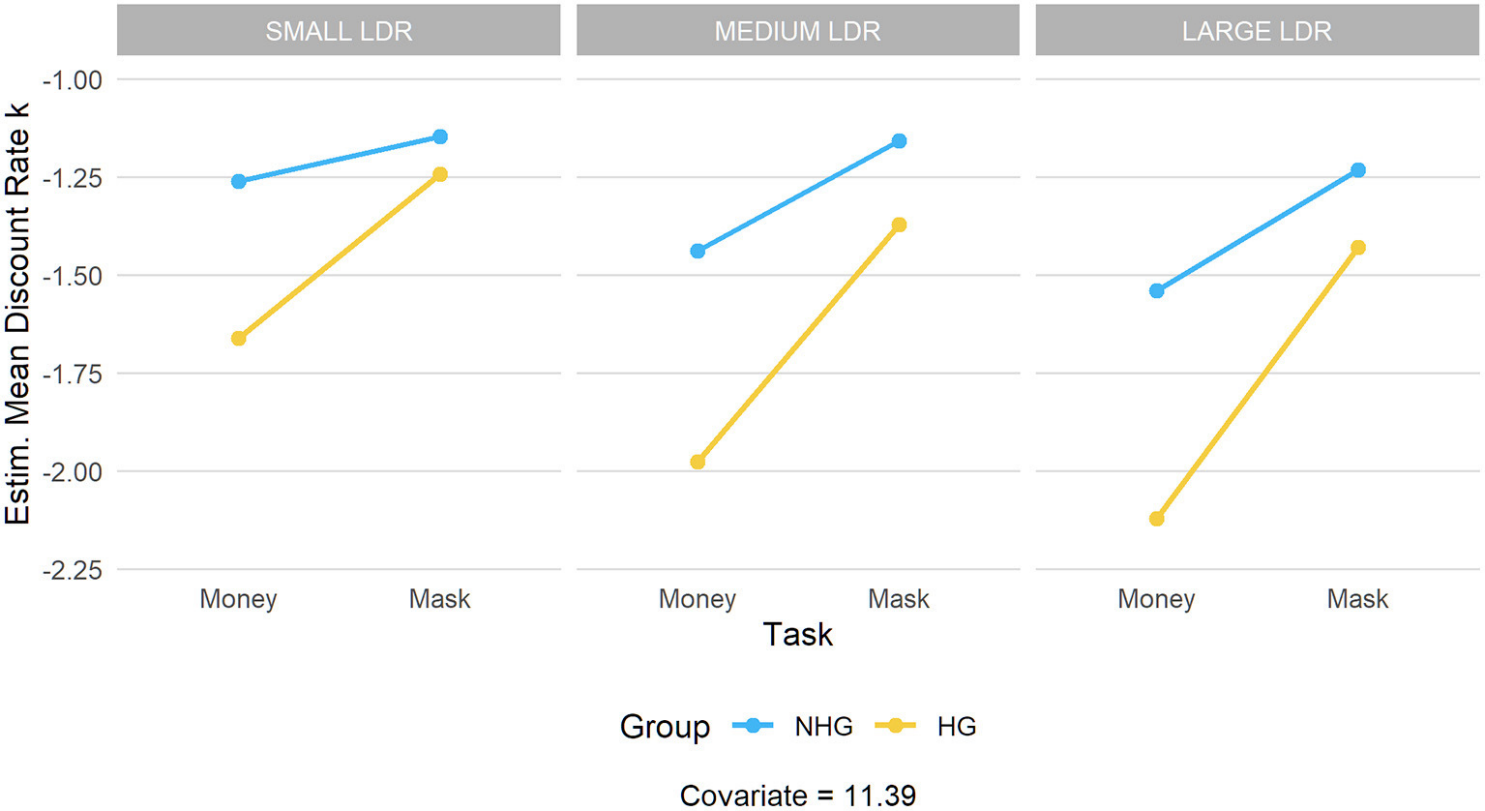
Estimated mean discount rate (small, medium, and large) across task versions (money vs. surgical mask) and hoarding group (HG vs. NHG).

The same ANCOVA was performed entering as dependent variable the discount rates *k* computed on the size (small, medium, and large) of the LDR. Results, confirming that the discounting pattern is quite similar across different LDR sizes and thus suggesting that no magnitude effect was detected, are reported in Figure 2.

At last, we performed Pearson correlations on the whole sample to assess the relationship between money discount rate, surgical masks discount rate, and hoarding dimensions (cluttering, difficult discarding, excessive acquisition, distress, impairment, and total HRS-I). As shown in Table 3, money and surgical masks discount rate were positively correlated (*r* = 0.20, *p* < 0.05). Moreover, significant correlations were found between each hoarding dimension and discount rate *k* in the money version of the task. On the other side, the discount rate in the surgical mask version of the task was found to be significantly correlated only with the cluttering dimension (*r* = −0.23, *p* = 0.01).

**Table 3:** Pearson's correlations between hoarding dimensions and discount rate (overall) in the money (*k* money) and masks version (*k* masks) of the discounting task.

**Variable**	**1**	**2**	**3**	**4**	**5**	**6**	**7**	**8**
1. Cluttering	–							
2. Difficult discarding	0.527^***^	–						
3. Excessive acquisition	0.520^***^	0.605^***^	–					
4. Distress	0.451^***^	0.365^***^	0.359^***^	–				
5. Impairment	0.476^***^	0.247^**^	0.350^***^	0.745^***^	–			
6. Total HRS-I	0.770^***^	0.755^***^	0.744^***^	0.775^***^	0.729^***^	–		
7. *k* (money)	−0.291^**^	−0.463^***^	−0.349^***^	−0.387^***^	−0.236^**^	−0.469^***^	–	
8. *k* (masks)	−0.234^**^	−0.143	0.061	−0.103	−0.053	−0.127	0.198^*^	–

* *p < 0.05*;

** *p < 0.01*;

*** *p < 0.001 (one-tailed)*.

## Discussion

In the current study, we examined the role of individual hoarding level on temporal discounting of money and surgical masks (a protective equipment for preventing the spread of COVID-19) during the peak of the COVID-19 Italian pandemic (March and April 2020). The main aim was to evaluate how an informational frame conveying a message of scarcity of surgical masks impacted intertemporal choices of hoarders and non-hoarders. Mixed model ANCOVA was used to investigate the effects of individuals' hoarding level on discount rate k using two versions of the Delay Discounting Task (money vs. surgical mask). The results highlighted a main effect of group with non-hoarders reporting higher discount rate than hoarders, regardless of the task type. Also, there was a main effect of task type since that, regardless of the individuals' hoarding levels, all participants showed a higher discount rate in the surgical masks version compared to the money version of the task. In other words, individuals showed a higher preference for an immediate reward rather than a delayed but larger in the surgical mask version, indicating more impulsivity when choosing on masks. A possible explanation relies on the influence of mortality salience during the surgical mask version of the task. Hence, there is evidence suggesting that mortality cues can increase the applied temporal discounting (e.g., Zaleskiewicz et al., 2013) and that neural representation of death thoughts/mortality in the cortical midline structures underpins this behavioral effect (Yanagisawa et al., 2021).

Noteworthy, NHG reported this effect more than HG. This surprising result becomes understandable when considering the theoretical framework suggesting that scarcity leads to “myopia” (short-sighted decisions), as it reduces attentional focus and consideration of future consequences (e.g., Tanaka et al., 2010; Haushofer and Fehr, 2014). While several studies confirmed this perspective, questionability of some of these results as well as divergent findings also exist (Camerer et al., 2018). For example, scarcity has been shown to induce people to consider opportunity-cost ratio and a general greater planning orientation to organize a strategy to cope with the shortage of commodities (e.g., Fernbach et al., 2015). Sharma et al. (2019) recently proposed a unifying approach for these mixed results. They indeed argued that scarcity does facilitate a reorientation but mainly in service of meeting an individual's most important need that may or not be near in time. Therefore, they proposed and demonstrated in a series of experiments that scarcity promotes decisions that favor the short-term exclusively when people perceive those choices as satisfying their essential needs. On the contrary, scarcity should lead to a farsighted decision when the option to wait to obtain a delayed and larger reward better answers the fulfillment of people's needs. This perspective fits well with our findings, in particular with the difference observed between hoarders and non-hoarders. A possible explanation may be that for non-hoarders the belief of a shortage in surgical masks' availability, together with the awareness that the use of masks was mandatory and needed to prevent the spreading of COVID-19, induced an orientation toward the need to obtain surgical masks as soon as possible. For non-hoarders, instead, the emergency may have also activated a second important need, namely the need to stock the commodity. Therefore, hoarders would undergo a double effect induced by the pandemic: one that directs the choice toward immediate options (as for non-hoarders), and a second one that pushes the choice toward delayed options to fulfill the need to plan and organize the stock for future use. Moreover, our result of a significantly lower money discount rate in HG is also in line with previous research that showed how, when using an implicit measure of impulsivity, higher discount scores were associated with lower hoarding symptom severity (Levy et al., 2019).

Altogether these results confirmed that a COVID-19 related commodity, that has been revealed to be in shortage, has been discounted significantly more than money. These results support those suggesting that scarcity anchors people's decision in the “here and now.” Moreover, our results are in line with a recent perspective on hoarding behavior based on a future-oriented mindset rather than an impulsivity-based explanation. Based on this perspective, the willingness of not being caught unprepared assumes a key role in hoarding behavior during uncertainty situations. Our study also presents some limits. First, we administered no explicit impulsiveness measures, that would have helped to further disambiguate the effect between hoarders and non-hoarders and would have facilitated the comparison between our data and previous studies. Second, the presence of a “middle” HG could have helped disentangle the obtained results. Third we did not include a third (control) version such as a temporal discounting task presenting a COVID-19 related commodity that was not immediately necessary (as it was the case for the surgical masks). This other condition would have helped to corroborate the theoretical frame proposed by Sharma et al. (2019) and represents a following step to be pursued in future works.

## Data Availability Statement

The datasets generated during and/or analyzed the current study are available in the Open Science Framework repository https://osf.io/h2vuf/?view_only=9629e9a0c6394c9d81e9e9444e32409b.

## Ethics Statement

The studies involving human participants were reviewed and approved by Institutional Review Board of Psychology (IRBP) of the Department of Psychological, Health and Territorial Sciences at G. d'Annunzio University of Chieti-Pescara. The patients/participants provided their written informed consent to participate in this study.

## Author Contributions

LC and SA conceived the experiment. LC, SA, AB, and ADC prepared tasks and conducted the experiment. LC and SA performed statistical analysis and figure generation. LC, RoP, IC, ADD, and RiP prepared the draft manuscript. All authors contributed to the article and approved the submitted version.

## Conflict of Interest

The authors declare that the research was conducted in the absence of any commercial or financial relationships that could be construed as a potential conflict of interest.
