# Supplementary material for: Temporal Discounting of Money and Face Masks During the COVID-19 Pandemic: The Role of Hoarding Level
Source: Front Psychol. 2021 Jun 9;12:642102. doi: 10.3389/fpsyg.2021.642102 (PMC8219851; doi:10.3389/fpsyg.2021.642102)
Supplement: Supplementary file 1 [file Data_Sheet_1.docx]

Supplementary Material

Hoarding Rating Scale

Please use the following scale when answering items below:

0 = no problem; 2 = mild problem, occasionally (less than weekly) acquires items not needed, or acquires a few unneeded items; 4 = moderate, regularly (once or twice weekly) acquires items not needed, or acquires some unneeded items; 6 = severe, frequently (several times per week) acquires items not needed, or acquires many unneeded items; 8 = extreme, very often (daily) acquires items not needed, or acquires large numbers of unneeded items.

1. Because of the clutter or number of possessions, how difficult is it for you to use the rooms in your home?

2. To what extent do you have difficulty discarding (or recycling, selling, giving away) ordinary things that other people would get rid of?

3. To what extent do you currently have a problem with collecting free things or buying more things than you need or can use or can afford?

4. To what extent do you experience emotional distress because of clutter, difficulty discarding or problems with buying or acquiring things?

5. To what extent do you experience impairment in your life (daily routine, job / school, social activities, family activities, financial difficulties) because of clutter, difficulty discarding, or problems with buying or acquiring things?

**Surgical mask version of the task**

The table S1 reports items used in the mask version of the task. Later Delayed Reward (LDR) Size, k at indifference (discount rate that participants should have to express indifference between the two options) and k rank (item rank as based on k at indifference) columns’ information were not available for participants.

**Instructions:** We ask you to perform a brief choice task. You will be presented with a series of choices between two options, one smaller but immediately available and one larger but delayed in time. For example, you will be asked if you would prefer to receive 10 single-use masks now or 15 single-use masks in 7 days. For each choice, indicate which of the two alternatives you prefer. Remember that there are no right or wrong answers.

**Table S1.**

| **Item** | **Smaller Option** | **Time of the Smaller Option** | **Larger Option** | **Time of the Larger Option** | **Later Delayed Reward (LDR) Size** | **k at indifference** | **k rank** |
| --- | --- | --- | --- | --- | --- | --- | --- |
| 1 | 54 single-use masks | now | 55 single use masks | 117 days | Medium | .00016 | 1 |
| 2 | 55 single-use masks | now | 75 single-use masks | 61 days | Large | .0060 | 5 |
| 3 | 19 single-use masks | now | 25 single-use masks | 53 days | Small | .0060 | 5 |
| 4 | 31 single-use masks | now | 85 single-use masks | 7 days | Large | .25 | 9 |
| 5 | 14 single-use masks | now | 25 single-use masks | 19 days | Small | .041 | 7 |
| 6 | 47 single-use masks | now | 50 single-use masks | 160 days | Medium | .00040 | 2 |
| 7 | 15 single-use masks | now | 35 single-use masks | 13 days | Small | .10 | 8 |
| 8 | 25 single-use masks | now | 60 single-use masks | 14 days | Medium | .10 | 8 |
| 9 | 78 single-use masks | now | 80 single-use masks | 162 days | Large | .00016 | 1 |
| 10 | 40 single-use masks | now | 55 single-use masks | 62 days | Medium | .0060 | 5 |
| 11 | 11 single-use masks | now | 30 single-use masks | 7 days | Small | .25 | 9 |
| 12 | 67 single-use masks | now | 75 single-use masks | 119 days | Large | .0010 | 3 |
| 13 | 34 single-use masks | now | 35 single-use masks | 186 days | Small | .00016 | 1 |
| 14 | 27 single-use masks | now | 50 single-use masks | 21 days | Medium | .041 | 7 |
| 15 | 69 single-use masks | now | 85 single-use masks | 91 days | Large | .0025 | 4 |
| 16 | 49 single-use masks | now | 60 single-use masks | 89 days | Medium | .0025 | 4 |
| 17 | 80 single-use masks | now | 85 single-use masks | 157 days | Large | .00040 | 2 |
| 18 | 24 single-use masks | now | 35 single-use masks | 29 days | Small | .016 | 6 |
| 19 | 33 single-use masks | now | 80 single-use masks | 14 days | Large | .10 | 8 |
| 20 | 28 single-use masks | now | 30 single-use masks | 179 days | Small | .00040 | 2 |
| 21 | 34 Single-use masks | now | 50 single-use masks | 30 days | Medium | .016 | 6 |
| 22 | 25 single-use masks | now | 30 single-use masks | 80 days | Small | .0025 | 4 |
| 23 | 41 single-use masks | now | 75 single-use masks | 20 days | Large | .041 | 7 |
| 24 | 54 single-use masks | now | 60 single-use masks | 111 days | Medium | .0010 | 3 |
| 25 | 54 single-use masks | now | 80 single-use masks | 30 days | Large | .016 | 6 |
| 26 | 22 single-use masks | now | 25 single-use masks | 136 days | Small | .0010 | 3 |
| 27 | 20 single-use masks | now | 55 single-use masks | 7 days | Medium | .25 | 9 |

**Money version of the task**

The table S2 reports items used in the money version of the task. Later Delayed Reward (LDR) Size, k at indifference (discount rate that participants should have to express indifference between the two options) and k rank (item rank as based on k at indifference) columns’ information were not available for participants.

**Instructions**: We ask you to perform a brief choice task. You will be presented with a series of choices between two options, one smaller but immediately available and one larger but delayed in time. For example, you will be asked if you would prefer to receive 10 € now or 15 € in 7 days. For each choice, indicate which of the two alternatives you prefer. Remember that there are no right or wrong answers.

**Table S2**.

| **Item** | **Smaller Option** | **Time of the Smaller Option** | **Larger Option** | **Time of the Larger Option** | **Later Delayed Reward (LDR) Size** | **k at indifference** | **k rank** |
| --- | --- | --- | --- | --- | --- | --- | --- |
| 1 | 54 € | now | 55 € | 117 days | Medium | .00016 | 1 |
| 2 | 55 € | now | 75 € | 61 days | Large | .0060 | 5 |
| 3 | 19 € | now | 25 € | 53 days | Small | .0060 | 5 |
| 4 | 31 € | now | 85 € | 7 days | Large | .25 | 9 |
| 5 | 14 € | now | 25 € | 19 days | Small | .041 | 7 |
| 6 | 47 € | now | 50 € | 160 days | Medium | .00040 | 2 |
| 7 | 15 € | now | 35 € | 13 days | Small | .10 | 8 |
| 8 | 25 € | now | 60 € | 14 days | Medium | .10 | 8 |
| 9 | 78 € | now | 80 € | 162 days | Large | .00016 | 1 |
| 10 | 40 € | now | 55 € | 62 days | Medium | .0060 | 5 |
| 11 | 11 € | now | 30 € | 7 days | Small | .25 | 9 |
| 12 | 67 € | now | 75 € | 119 days | Large | .0010 | 3 |
| 13 | 34 € | now | 35 € | 186 days | Small | .00016 | 1 |
| 14 | 27 € | now | 50 € | 21 days | Medium | .041 | 7 |
| 15 | 69 € | now | 85 € | 91 days | Large | .0025 | 4 |
| 16 | 49 € | now | 60 € | 89 days | Medium | .0025 | 4 |
| 17 | 80 € | now | 85 € | 157 days | Large | .00040 | 2 |
| 18 | 24 € | now | 35 € | 29 days | Small | .016 | 6 |
| 19 | 33 € | now | 80 € | 14 days | Large | .10 | 8 |
| 20 | 28 € | now | 30 € | 179 days | Small | .00040 | 2 |
| 21 | 34 € | now | 50 € | 30 days | Medium | .016 | 6 |
| 22 | 25 € | now | 30 € | 80 days | Small | .0025 | 4 |
| 23 | 41 € | now | 75 € | 20 days | Large | .041 | 7 |
| 24 | 54 € | now | 60 € | 111 days | Medium | .0010 | 3 |
| 25 | 54 € | now | 80 € | 30 days | Large | .016 | 6 |
| 26 | 22 € | now | 25 € | 136 days | Small | .0010 | 3 |
| 27 | 20 € | now | 55 € | 7 days | Medium | .25 | 9 |

**Post task survey**

1. Indicate your age
2. Indicate your gender
3. In your opinion, how long will it take to return to normal life (where normal means that you can do everything you did before COVID-19 pandemic)? Express your answer in months.
